# Alloantibodies and Drug-Induced Immune Hemolytic Anemia in Liver Transplantation

**DOI:** 10.1155/2011/250674

**Published:** 2011-06-30

**Authors:** Surekha Devi Allanki

**Affiliations:** Department of Transfusion Medicine, Global Hospitals, Hyderabad 500004, India

## Abstract

Red blood cell alloantibodies can cause severe delayed hemolysis, despite immunosuppression which they receive posttransplantation. A female patient with Hepatitis C-related chronic liver disease reported to our center for liver transplantation. During preoperative evaluation, she was found to have significant red blood cell alloantibodies which gave rise to problems during pre transfusion compatibility test. Stringent measures were taken by the transplant team to minimize blood loss during surgery. It was decided to have lower transfusion trigger for red cell transfusion, and blood conservation was done by intraoperative red cell salvage and use of antifibrinolytic agent. During immediate postoperative period, she developed drug-related immune hemolytic anemia. Presence of both warm autoantibodies and alloantibodies posed a big challenge for us to get cross-match compatible blood. She received 22 units of crossmatch compatible red cell transfusions during her hospital stay, which was uneventful. Hence, we reported this case.

## 1. Introduction

Clinically significant red blood cell (RBC) alloantibodies are present in 6% of liver transplant patients [1], due to sensitization through previous blood transfusions. 

Presence of significant RBC alloantibodies in these patients complicates the blood bank's ability to provide the necessary blood products [2]. Such patients pose challenge to transfusion services as they may require massive blood transfusions during liver transplantation, which is technically demanding procedure with associated hemostatic defects. 

## 2. Case Report

46-year-old female patient whose blood group was “A_1_ Positive” underwent deceased donor liver transplantation (DDLT) from identical blood group donor for end-stage liver disease due to Hepatitis C with MELD score of 26. Pre-operatively, she had comorbidities in the form of diabetes mellitus and recurrent hepatic hydrothorax requiring frequent therapeutic aspiration.

Past history revealed that she had three children underwent Caesarean section during third delivery. She received two units of blood transfusion during Caesarean section, and there was no history suggestive of transfusion reaction following blood transfusion. In 2005, she was admitted in outside center for Hepatitis C-related liver disease. She received several units of fresh frozen plasma (FFP) and one unit of blood transfusion, which was followed by hemolytic transfusion reaction. Later, she did not receive any blood transfusions in other centers as cross-match was found to be incompatible. 

She was worked up for liver transplantation in our center in February 2006. During preoperative evaluation, Indirect Antiglobulin Test (IAT) was positive and Direct Antiglobulin Test (DAT) was negative. She was found to have significant alloantibodies (1 : 256), specificity could not be typed as there were multiple antibodies. There were no cold and warm autoantibodies. DAT, IAT, and compatibility test were done by column agglutination technique (CAT).

She was given erythropoietin regularly during waiting period for liver transplantation and maintained hemoglobin level of 10-11 gm%. Preoperative autologous deposit and plasma exchange (to lower titers of alloantibodies) were not done as she was waitlisted for DDLT.

She underwent DDLT in February 2007. We had limited inventory of cross-match compatible A Positive red cell units at the time of her surgery. Stringent measures were taken by the transplant team during surgery to minimize blood loss and allogenic blood transfusions. It was decided to have lower transfusion triggers; Hb < 6 gms% instead of <8 gm% for red cell transfusion, to go for intraoperative red cell salvage, and to give antifibrinolytics during surgery.

Intraoperatively, Tranexamic Acid (TA) was given in a dose of 10 mg/kg as bolus, followed by TA infusion (5 mg/kg/hr) throughout the surgery. She received 6 units of Cytomegalo virus (CMV-) reduced risk and leuco-reduced Antiglobulin cross-matched compatible red cell units, 6 units of FFP, 10 units of pooled cryoprecipitate, and 1 unit of CMV-reduced risk and leuco-reduced apheresed platelets during surgery, apart from 460 mL of salvaged blood which was reinfused. Intraoperatively, there was no evidence of hemolysis. During surgery, she received Ceftizoxime 2 gm IV bolus followed by 1 gm IV tid which was continued in immediate postoperative period. Apart from this, she also received Levofloxacin and Flucanazole. She was given intravenous Heparin infusion intraoperatively for organized portal vein thrombosis. On day 1 of postoperative period, patient presented with hemolysis, which manifested as extra vascular hemolysis, positive DAT, clinically significant warm autoantibodies in a titer of >1 : 1024, increased reticulocyte count (2.2%), indirect bilirubin (3.3 mg/dL), and LDH (1278). After Ceftizoxime was withdrawn, clinically there was no evidence of hemolysis, reticulocyte count (0.8%), and LDH (320) dropped, but DAT remained positive, and titers of warm autoantibodies remained 1 : 1024 throughout her hospital stay. Alloantibodies titer dropped to 1 : 8 during postoperative period, which was probably due to the immunosuppressive drugs (Mycophenolate and Tacrolimus) which she received during postoperative period. She underwent reexploration on day 6, clot evacuated and hemostasis achieved. Later, she developed fungal sepsis due to mucormycosis and she was on liposomal Amphotericin B. She received 22 units of Antiglobulin cross-match compatible red cell units during postoperative period, which was uneventful.

## 3. Discussion

In this patient, alloantibodies were probably pregnancy induced, due to anamnestic response which caused the appearance of IgG antibodies that reacted with the transfused red cells, thereby leading to hemolytic transfusion reaction in the past. 

Second and third generation Cephalosporins are responsible for most cases of drug-induced immune hemolytic anaemia (DIHA) [3]. DIHA is attributed most commonly to drug-dependant antibodies that can only be detected in the presence of drug [4]. The drug covalently binds to proteins (e.g., RBC membrane proteins), RBCs become coated with drug in vivo and a drug antibody (usually IgG) attaches to the drug-coated RBCs that are subsequently cleared by macrophages [5]. Based on the clinical evidence of association of hemolysis with initiation of the drug, Ceftizoxime and resolution of symptoms with discontinuation of the drug in this patient, the possibility of DIHA was thought of. Paradoxically, this patient had received the same drug in the past and it was uneventful. Probably due to reactivation of pre-existing sensitization, drug-related warm autoantibodies developed in this patient. 

If the alloantibody is identified, corresponding antigen-negative red cell units are transfused. In this patient, as specificity of antibodies could not be done (because they were multiple), Antiglobulin cross-match compatible red cell units were transfused. 

The incidence of alloimmunization varies with ethnic heterogeneity of the population. Luzo et al. reported 23% incidence of red cell alloantibodies among liver transplantation patients in their center [6]. Ramsey et al. reported that out of 1000 liver transplantations performed in their center, 13.7% of adults and 6.3% of children had significant RBC alloantibodies [2]. A retrospective study done by Au et al. showed 17 patients (8.8%) of RBC alloantibodies in 192 consecutive Chinese recipients of liver transplants [7]. Hareuveni et al. reported a case of delayed, anti-JK(a)-mediated hemolysis in a liver transplant recipient, caused by passenger donor lymphocytes [8]. Blood banks servicing transplant centers should be aware of ethnic patterns in RBC alloantibodies. 

## 4. Conclusion

Providing cross-match compatible blood to a patient with significant alloantibodies and warm autoantibodies during liver transplantation and immediate postoperative period posed a big challenge to Transfusion Services. All blood transfusions were uneventful. Hence, we reported this case.

Delayed hemolysis may jeopardize patient survival as a result of difficult postoperative stabilization, especially in patients requiring massive/multiple transfusions. Resolution of these problems is an important part of Blood Transfusion Services, which is necessary for a liver transplant program.
